# From home energy management systems to energy communities: methods and data

**DOI:** 10.1038/s41597-024-03184-5

**Published:** 2024-04-06

**Authors:** Antonio Ruano, Maria da Graça Ruano

**Affiliations:** 1 IDMEC, Instituto Superior Técnico, Universidade de Lisboa, Lisboa, and Faculty of Science & Technology, University of Algarve, Faro, Portugal; 2 CISUC, University of Coimbra, Coimbra, and Faculty of Science & Technology, University of Algarve, Faro, Portugal

**Keywords:** Energy grids and networks, Photovoltaics

## Abstract

This paper introduces the HEMStoEC database, which contains data recorded in the course of two research projects, NILMforIHEM, and HEMS2IEA, for more than three years. To be manageable, the dataset is divided in months, from January 2020 until February 2023. It consists in: (a) consumption electric data for four houses in a neighbourhood situated in the south of Portugal, (b) weather data for that location, (c) photovoltaic and battery data, (d) inside climate data, and (e) operation of several electric devices in one of the four houses. Raw data, sampled at 1 sec and 1 minute are available from the different sensing devices, as well as synchronous data, with a common sampling interval of 5 minutes are available. Gaps existing within the data, as well as periods where interpolation was used, are available for each month of data.

## Background & Summary

Over the last two decades the global electricity consumption market has been growing at an average yearly reported level of 3.1%. One of the largest consumer sector are buildings, and in particular the residential sector. Managing efficiently the flow of electricity in a house is important, not only from the point of view of the owner’s electricity bill, but also from the point of view of global consumption, as well as from the point of view of the electrical grids. In fact, traditional grids find it difficult to cope with this increasing demand, exacerbated by the integration of extensive variable energy resources, such as renewable energy systems.

The present dataset is the result of two projects NILMforIHEM, and HEMS2IEA. The aims of the first project were to improve the performance of existing non-intrusive load monitoring algorithms and the efficiency of energy systems in homes. The second project, using the results of the former, aimed to propose new energy management techniques for local energy communities, managed by an aggregator. It was considered that the aggregator would interface with each residential management system and with the electricity grid, allowing electricity to be managed in accordance with different community contracts. The dataset enables several different topics related to the efficient use of energy in households and communities to be investigated by the research community. In the sequel a brief review of these topics is conducted.

### Home energy management systems

The goal of a Home Energy Management System (HEMS) is to manage efficiently the flow of electricity in the house, so that the electric bill is reduced or annulated, maintaining the comfort of its occupants. Despite the large interest of the research community, due to the complexity and diversity of the systems, as well as by the use of suboptimal control strategies, energy consumption is still higher than necessary, and users are unable to yield full comfort in their homes. Excellent reviews detailing HEMS developments in recent years are available; please consult the reviews of Beuadin and Zareipour^1^, Leitão and co-workers^2^, Mahapatra Mahapatra and Nayyar^3^ or Gomes *et al*.^4^. According to this last reference, HEMS can be broadly divided into four classes: traditional techniques, model predictive control, also known as model-based predictive control (MBPC), heuristics and metaheuristics, and other techniques. The first class comprises methods based on traditional optimization techniques, typically using commercial solvers. Perhaps the most important sub-class within traditional methods is the use of Mixed-Integer Linear Programming (MILP), which refers to optimization techniques where the objective function is a linear function and subject to linear restrictions, but includes mixed, continuous and discrete variables. Examples of household energy management based on MILP are the works of:

- Lu *et al*.^5^, where the results of the proposed HEMS are compared with other energy management systems, showing the effectiveness of the proposed model, through case studies that allow reducing energy costs in both summer and winter;
- Baek *et al*.^6^, where results are compared when demand response is employed and when it is not. They demonstrate that the strategy presented with demand response is superior;
- Lyu *et al*.^7^, where the proposed methodology allows to reduce house costs by 53% and reduce Peak-to-Average Ratio (PAR) by around 70%.

Model-based predictive control is an advanced control technique based on a receding horizon principle, aimed at determining the best sequence of actions while meeting the requirements. The application of MBPC in HEMS has increased significantly in recent years. For instance, in Mirakhorli *et al*.^8^ a HEMS for a residential building with a Photovoltaic (PV) system, Electric Storage System (ESS), thermal and electric loads, and Electric Vehicles (EV) is proposed. The MBPC problem considered a prediction horizon of four hours for every five minutes. Rao and co-workers^9^ propose a HEMS for a smart home focusing on the energy balance between the three phases to control both active and reactive power. Several case studies are considered, assuming a prediction horizon of twenty-four hours, a control horizon of twenty-four hours, and a simulation horizon of forty-eighty hours. A comprehensive approach of a mixed-integer quadratic-programming MPC scheme based on the thermal building model and the building energy management system is employed by Killian and co-workers^10^.

### Heating, ventilation and air conditioning systems

It is recognized that near 40% of the energy (see Pérez-Lombard and co-workers^11^) consumed in buildings is due to the operation of Heating, Ventilation and Air Conditioners (HVAC). For this reason a special care should be devoted to this specific equipment. MBPC is perhaps the most proposed technique for HVAC control since it offers an enormous potential for energy savings. Typically what is sought is the minimization of the energy spent, or the electricity bill, incurred in the HVAC operation, while simultaneously maintaining the room(s) under thermal comfort. Thermal comfort can be assessed in different ways, the most used being temperature regulation. In some cases, the relative humidity is also maintained within user-defined bounds. In the last years, the Predicted Mean Vote (PMV) is increasingly used. The PMV index is based on human thermal sensation which is strongly related with the energy balance of the body when the human body is considered in a heat balance situation, i.e., the heat produced by metabolism equals the net loss of heat. The classical way in which the PMV index can be obtained was presented by Fanger^12^ and is dependent on six variables: metabolic rate, clothing insulation air temperature, relative humidity and velocity, and mean radiant temperature.

For HVAC control, MBPC can be applied in several different ways. Donaisky and co-workers^13^ minimized the PMV index, generating a nonlinear PMV model having a Wiener structure. Ma *et al*.^14^ employ a simple thermal mass model to minimize a cost function employing economic costs. Castilla *et al*.^15^ minimize the PMV index, using a PMMPC model. In Chen’s work^16^ the energy is (indirectly) minimized, using constraints on the thermal sensation scale, where the use of the PMV index is compared with an Actual Mean Vote index. A simple thermal model is used in this approach. In Huang *et al*.^17^ a neural network is used to optimize a start-stop strategy for temperature-regulated control. Li *et al*.^18^ minimize the energy spent and violations of bounds on air temperature, using a state-space formulation for the prediction of these variables.

### Non-intrusive load monitoring

Energy monitoring is a key point of a HEMS; it can be done installing measuring devices at every load of interest or using Non-Intrusive Load Monitoring (NILM) methods, which disaggregate the overall usage, using a measure of the load at the utility service entry. Research, however, is still needed in this field, specially in terms of simple algorithms, without requiring either special-purpose hardware or the use of high-sampling power data.

Excellent reviews on NILM algorithms can be found in the works of Georgios Angelis *et al*.^19^ and Ruano and co-workers^20,21^.

The main stages in a NILM application are^21^:

- Data collection: electrical data, including current, voltage, and power data, are obtained from smart meters, acquisition boards or by using specific hardware;
- Event detection: an event is any change in the steady state of an appliance over time. An event implies variations in power and current, which can be detected in the electrical data previously collected by means of thresholds;
- Feature extraction: appliances provide load signature information or features that can be used to distinguish one appliance from another;
- Load identification: using the features previously identified, a classification procedure takes place to determine which appliances are operating at a specified time or period, and/or their states.

Regarding step (a), the most important point to consider is the sampling interval applied to the electrical signals. They can broadly be classified into *very low* (slower than one minute), *low* (between than one minute than one second), *medium* (sampling frequency between one and fifty/sixty Hz), *high* (from fifty/sixty Hz to two kHz), *very high* (between two and forty kHz) and *extremely high* (greater than forty kHz). Another point to take in consideration is the hardware used to acquire the data. Commercial devices typically only achieve very low and low frequencies; higher sampling frequencies need specialized hardware. Related with that are data storage and processing capabilities, which obviously increase with the sampling frequency employed.

Focusing now at step (b), according to the work of Anderson *et al*.^22^ event detectors typically use three different approaches: expert heuristics, probabilistic models and matched filters. The former consist of the creation of a set of rules for each appliance. Initial NILM works used this approach. Probabilistic models provide a probability, used to make a decision about the occurrence of events. A particularly well-known case is the Generalized Likelihood Ratio (GLR) method (please see Anderson’s work^22^). Finally, matched filters are characterized by extracting the signal waveforms and correlating them with known patterns.

The features that can be used to identify an appliance are obviously related to the sampling time employed. For very low and low frequencies, active, apparent and reactive powers are often used, together with Root-Mean-Square values of the current or voltage. Medium rate acquisition allows the use of transient features of the electrical features. High sampling rates allow to employ spectral features such as harmonics (see Meehan *et al*. work^23^), Discrete-Wavelet Transform (Chang and co-workers^24^), and so on. Very high rate data allows to obtain much more detail about each appliance’s waveform, either from the higher harmonics or from the shape of the raw current and voltage waveforms themselves. Two-dimensional voltage-current (V-I) trajectories were used in Hassan and co-workers investigation^25^.

Using the features described above, computed from the aggregate load, the objective in step d) is to identify the appliances that are operating at a given time. This can be formulated as a optimization or classification problem, as four appliance types are usually considered:

- Type I—On/off devices: most appliances in households, such as bulbs and toasters;
- Type II—Finite-State-Machines (FSM): the appliances in this category present states, typically in a periodical fashion. Examples are washer/dryers, refrigerators, and so on;
- Type III—Continuously Varying Devices: the power of these appliances varies over time, but not in a periodic fashion. Examples are dimmers and tools.
- Type IV—Permanent Consumer Devices: these are devices with constant power but that operate 24 h, such as alarms and external power supplies.

This way, for the case of type II appliances, identification is not only translated into which appliances are active, but also their states.

A very large number of techniques have been proposed for this step. They can be very broadly classified as optimization methods and machine learning (supervised and unsupervised) techniques. Optimization approaches use different methods to perform a combinatorial search. Examples are hybrid programming (Kong *et al*. work^26^), genetic algorithms (Egarter, Sobe & Elmenreich paper^27^) and others. Supervised techniques use offline training to achieve a database of information used to design the classifier(s). These are the most employed class of methods; the works of Chang *et al*.^24^, Kelly & Knottenbelt^28^ and Wu and Wang^29^ belong to this class. Unsupervised methods do not require any training prior to classification, which is an important advantage. Feature clustering, and the later labelling of each cluster with meaningful appliance names has been applied by Yang and co-workers^30^. The most recent unsupervised techniques applied to NILM belong to a family of methods that assume that the electrical signal is the output of a stochastic system, maintaining a representation of the whole system state, instead of dealing with individual events. Examples are Hidden Markov Methods (HMM) and variants (please see the works of Cutsem *et al*.^31^ and Kong *et al*.^32^).

### Forecasting

Another important point for HEMS is the ability to forecast the values of important variables for energy management. And several forecasts are necessary, such as the home load demand, either global or appliance-based, the electricity produced by renewable energy sources, if available, weather variables, occupancy, inside climate, for instance. The better the quality of the estimation, the better the electricity management that can be achieved.

Forecasting techniques can be envisaged from several points of view, such as: (a) the time-scales involved; (b) the exogeneous variables used in the model; and (c) the methods applied. Regarding the former, time-scales can vary from horizons of a few seconds or minutes (intra-hour or very short forecasts, for control and adjustment actions), a few hours (intra-day or short/medium, for energy resource planning and scheduling, as well as for the electricity market), to a few days ahead (intra-week or long, for unit commitment and maintenance schedules). The choice of employing exogeneous variables, and in the affirmative case, which variables are used depends essentially on the model application. Finally, looking at the methods, in the general case they can be broadly divided into statistical and machine learning methods (obviously forecasting of specific variables may employ other class of methods). Statistical models are typically linear models such as persistent forecasts, Auto-Regressive (AR), Auto-Regressive–Moving-Average (ARMA), and Auto-Regressive Integrated Moving Average—ARIMA. Machine Learning methods are the most used nowadays and typically comprise several different shallow and deep neural networks, whether isolated or fusing different models.

Regarding PV power forecasting, several reviews exist in the topic. The interested reader can consult, for instance, the works of Alcañiz *et al*.^33^ or Pandžić and Capuder paper^34^, and the references within. Forecasting PV power will also need the forecasting of atmospheric variables, such as solar irradiation (please see El-Amarty *et al*. work^35^), air temperature (Tran *et al*.^36^), and possibly others. As examples, Yang and co-workers^37^ proposed a hybrid scheme, involving classification, training, and forecasting stages. This scheme is used for one-day ahead hourly forecasting of PV output. Fonseca and co-workers^38^ compare the suitability of a non-parametric distribution and three parametric distributions in characterizing prediction intervals for photovoltaic energy forecasts with high levels of confidence. Mei *et al*.^39^ propose an LSTM-Quantile Regression Averaging-based nonparametric probabilistic forecasting model for PV output power.

Households load demand forecasting is an active area of research as, on one hand, it allows the occupants to be aware of the energy consumption of their own house and, consequently, to take measures to reduce this consumption and the energy bill, and, on the other hand to enable a more efficient operation of the HEMS. During the last years, computational intelligence techniques somehow replaced physical-based methods, as the former do not require knowledge of the building geometry and physical phenomena to deduce an accurate prediction model. Several reviews exist on this topic, such as Foucquier’s^40^, Wei *et al*.^41^, Ahmad *et al*.^42^ and Wen *et al*.^43^. As in the case of PV forecasting, different exogenous variables can be applied to the prediction models, such as atmospheric air temperature, number of occupants, codifications of days between, week, weekend, and holidays, to name but a few. Different computational methods can also be applied. For instance, Mynhoff *et al*.^44^ compared different prediction models: Artificial Neural Networks-Nonlinear Auto-Regressive (ANN-NAR), HMMs, Support Vector Machines (SVM), MultiLayer Perceptrons (MLP) and Deep Belief Networks (DBN) for one-step daily and weekly forecasts. Yildiz and co-workers^45^ compared the forecasting performance of ANNs, SVMs and Least-Squares SVMs, with different data resolutions and forecasting horizons, with several models, each applied to a different load profile, obtained by clustering the load profiles.

Forecasts can also be applied to energy markets. In recent years, in many countries, the acquisition and sale of electricity is traded in energy markets (please see Yildiz and co-workers^46^). Accurate forecasts of the electricity demand and price are therefore a need for the participants in the energy markets. In particular, the one-day ahead hourly forecast, considered a short-term forecast, has received increasing attention from the research community. Comprehensive reviews on load and price forecasting are available in Suganthi & Samuel^47^ and Weron’s work^48^ respectively.

Finally, according to Zhang, He & Yang^49^, existing load and generation forecasting algorithms can be classified into two classes: point forecasts and probabilistic forecasts. The former provides single estimates for the future values of the corresponding variable, which are not capable of properly quantifying the uncertainty attached to the variable under consideration. The latter algorithms are increasingly attracting the attention of the research community due to their enhanced capacity to capture future uncertainty, describing it in three ways: prediction intervals, quantiles, and probability density functions (PDF) (please see Bracale and co-workers^50^).

### Communities of energy

Obviously, better and more efficient solutions, not only from each householder’ point of view, but also from the community consumption perspective, are extensions of the tools above described to groups of households that share between them the energy produced or stored, in the form of communities of energy. In this context the local HEMS can be hierarchically controlled by an aggregator, which supervises not only the management of energy in each local prosumer (productor/consumer), but also the flow of energy between the members of the community as a whole, as well as the exchanges between the community and the grid.

It is within this context that this dataset is introduced. It spans more than three years of data, covering different types of variables of high importance to the field of electrical energy and thermal comfort of, either isolated or community-based households. More specifically, it allows, for a single prosumer, to:

- Test and validate different control strategies for home energy management systems, as done by us in^51,52^. The first reference compares MBPC control implemented with the Branch-and-Bound technique for HVAC control with the house proprietary system. The second reference employs a MILP method in a MBPC framework, controlling not only the inverter, but also appropriately scheduling loads. Both approaches achieve important savings in the electricity bill.
- Design forecasting energy consumption models, as discussed in^53–55^. The first reference employs a design Multi-Objective-Genetic-Algorithm (MOGA)^56^ framework available in our lab, which performs feature selection, topology determination and parameter estimation, to forecast load demand forty-eight-steps-ahead, with a time-step of fifteen minutes. The second one extends the previous approach to an ensemble of MOGA designed models. The third one proposes an hybrid forecasting mechanism to use with^52^.
- Design forecasting PV energy generation models^57^. The approach described above is applied to PV power generation, with great success.
- Moving from deterministic forecasting to probability forecasting, for both load demand and PV power generation^58^
- Test and validate different non-invasive load monitoring (NILM) algorithms, as performed in^59,60^. The first reference employs ApproxHull^61^, a data selection tool existing in our lab to deep learning models. The second one uses ApproxHull and MOGA to design shallow models to detect appliance operation and energy estimation,
- Design forecasting thermal comfort models, as well as test and validate control strategies for Heating, Ventilation and Air Conditioning (HVAC) systems, as in^62^. Very basically, HVAC is controlled so that it guarantees PMV thermal comfort within user-predefined schedules, while minimizing the energy consumed, making use of forecasting models of solar radiation, atmospheric air temperature and relative humidity, inside air temperature, relative humidity and mean radiant temperature, as well as room occupancy.
  Additionally, for a community of four houses, it allows to:
- Test and validate different control strategies for the community energy management system, which can be found in^63^, where the MILP-MBPC strategy described above is extended for a community of houses. Different ways to share the produced and stored energy are compared.
- Design day-ahead net load point and probabilistic forecasting to work with energy markets, in^64^;
- Test and validate transfer learning strategies for NILM, as discussed in D’Incecco’s work^65^.

All the above topics are important, on their own, for future research. What perhaps is most important and should be stressed is that significant improvements on the general field of energy efficiency in buildings and energy communities require the join research of all these topics, to which others can obviously be added. This is an added-value of this dataset in comparison with existing ones, as this includes all the data needed to address all the topics considered, which is not verified in existing datasets.

As the households that were employed in this research are typical Mediterranean detached family houses, the data available in this dataset can be used as representative of that segment of buildings, and climate. By this we mean that it is expectable that methods and techniques applicable to the nine classes of problems identified above, using this dataset, will produce similar results to other households or communities in regions with a similar climatic type.

As both raw data, typically sampled at one second or at one minute (please see below) and curated data, synchronized with a five minutes sampling are available, different sampling intervals can be used for the different methods. The dataset can be found at^66^.

## Methods

Data was collected from four residential houses, situated in Gambelas, Faro, in the south of Portugal. All four are detached houses, with two floors and garden, where families live. Two of the houses have triphasic meters, while the others are monophasic. The former will be denoted as TH1 and TH2, while the latter are coined MH1 and MH2. TH1 has a PV system and a energy storage, MH1 has a photovoltaic system, and the others do not have any renewable energy source.

TH1 was used in NILMforIHEM project, that started in 2019. For this reason, and because it was used for objectives a) to f) above, has much more data for a much larger period of time. This house and the three additional houses were employed for project HEMS2IEA, which started in 2021. Only electric consumption data was recorded for these three houses. Recorded data for the four houses spans from November 2021 until July 2022. After this date, as one of the houses had major works, data was reduced to three houses.

**Fig. 1:**
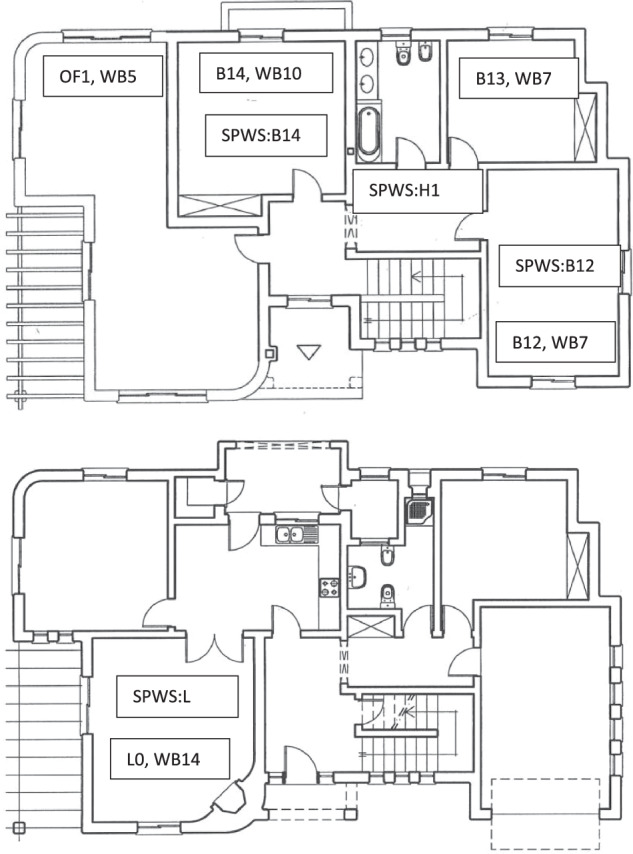
Floor plans of TH1. Top: 1^st^ floor; bottom: ground floor.

TH1 has twenty different spaces (including garden, halls, and so on). The floor plans are shown in Fig. 1.

**Fig. 2:**
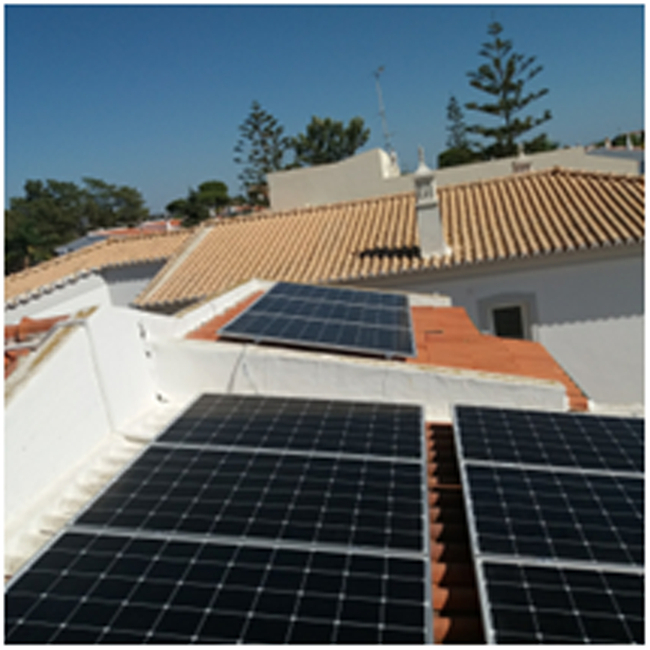
Photovoltaic panels.

**Fig. 3:**
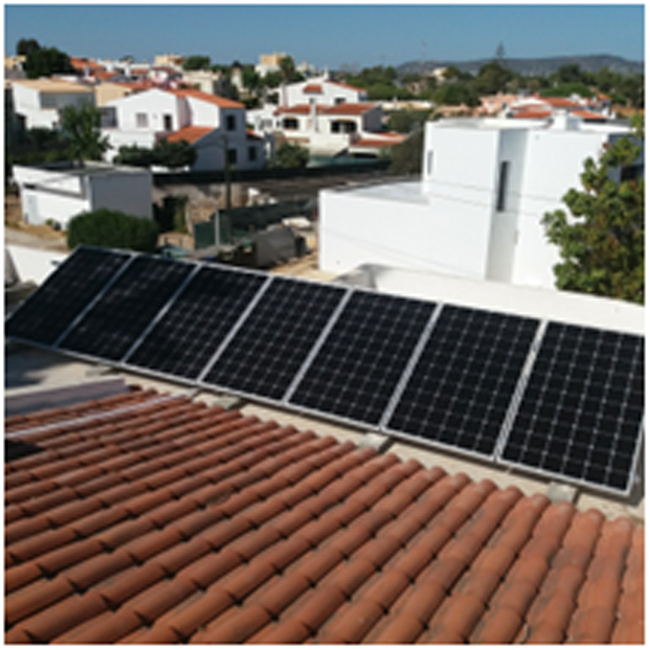
Photovoltaic panels.

**Fig. 4:**
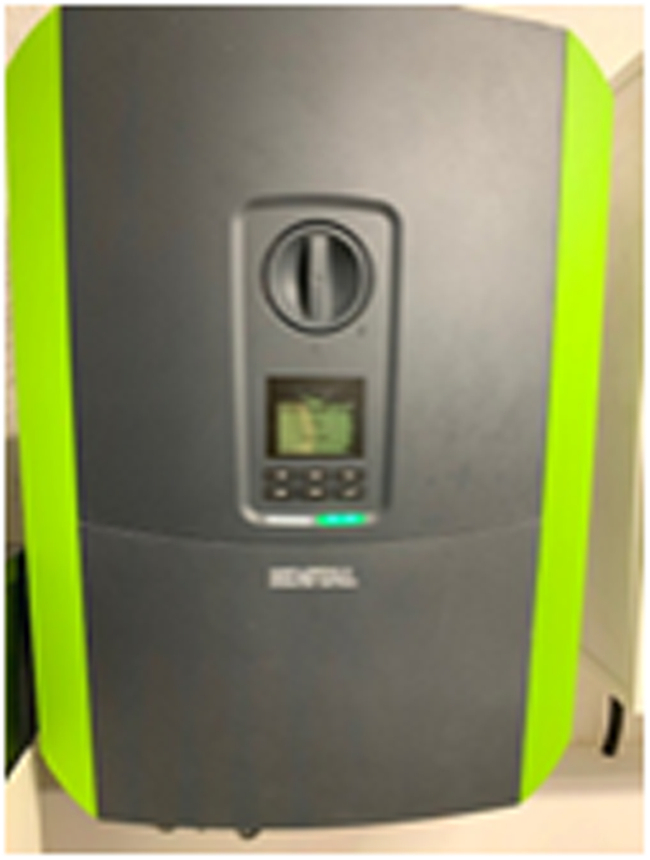
Inverter.

**Fig. 5:**
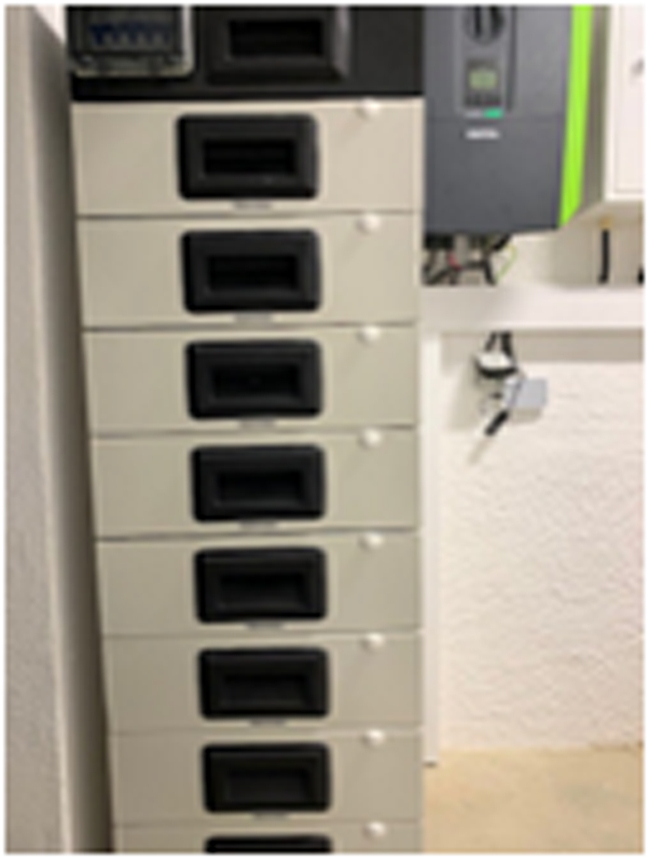
Battery Box.

A photovoltaic system was installed, composed of 20 Sharp NU-AK panels^67^, each panel with a maximum power of 300 W. (please see Figs. 2 and 3) The inverter is a Kostal Plenticore Plus (Fig. 4) converter (KI)^68^, which also controls a BYD Battery Box (Fig. 5) HV H11.5 (with a storage capacity of 11.5 kWh)^69^.

The house electric panel consists of sixteen monophasic circuit breakers, plus a triphasic one. Several electric variables are measured in every circuit breaker, providing approximate ground truth for the NILM identification. Circutor Wibees (WB)^70^ are used as the measurement devices. They are plug and play wireless devices and use Hall Effect technology for the measurement. Because of that, calibrations are required for correct measurements. Voltage, current, frequency, active reactive and apparent power, power factor, active inductive reactive and capacitive reactive energy are measured every second for the every monophasic circuit breakers, the same number for each phase of the triphasic one, together with totalized values. In total, 198 variables are sampled by the WBs every second.

Total consumption data is supplied by a Carlo Gavazzi (EM340) three-phase energy meter^71^. This meter is a class X certificated device, and electrical measurement is done using a two-wires Modbus RTU connection. EM340 supplies 37 different electric variables, sampled at one Hz.

Measurements of the energy produced by the PV, stored in the battery and injected in the grid are obtained either from the inverter (KI) or from a Kostal smart energy meter (KEM)^72^. Home electrical consumption variables are also available in the inverter. In total, 78 variables are obtained by KEM and KI, at a sampling interval of one minute (Fig. 9).

**Fig. 6:**
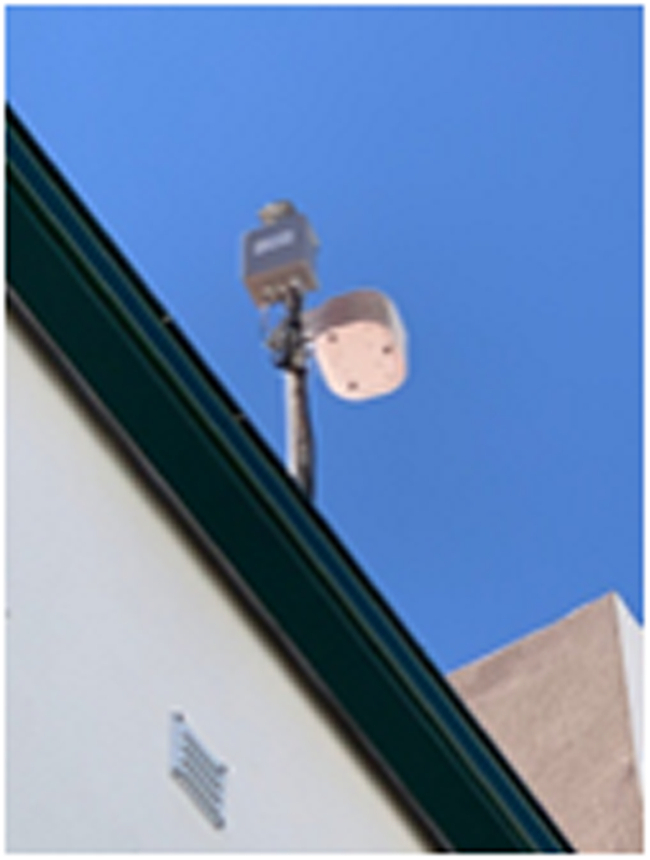
Weather Station.

**Fig. 7:**
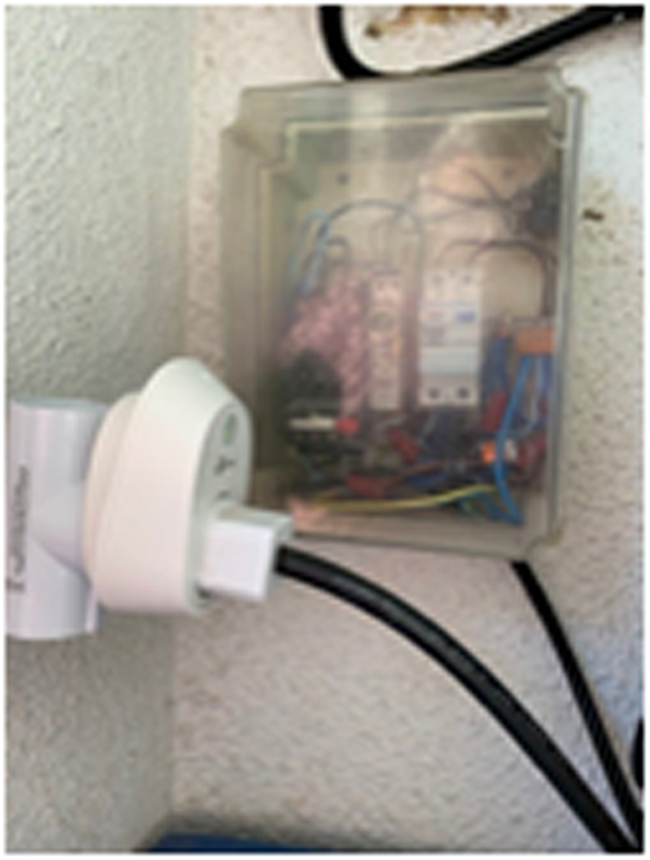
Smart Plug.

For on/off control Smart Plugs Self-Powered Wireless Sensors^73^ are used (Fig. 7). They are also used to enable sockets belonging to the same CB to be measured individually. They are read/controlled directly using an internal web service. The number of SPs changed with time, enabling the measurement of six variables every second for each plug. In a similar way to the SPs, the Air Conditioner in Room B14 in Fig. 1 can be measured and actuated.

A Weather station (please see Mestre *et al*.^74^) measures the air temperature and relative humidity, and global solar radiation, at one second intervals (Fig. 6).

**Fig. 8:**
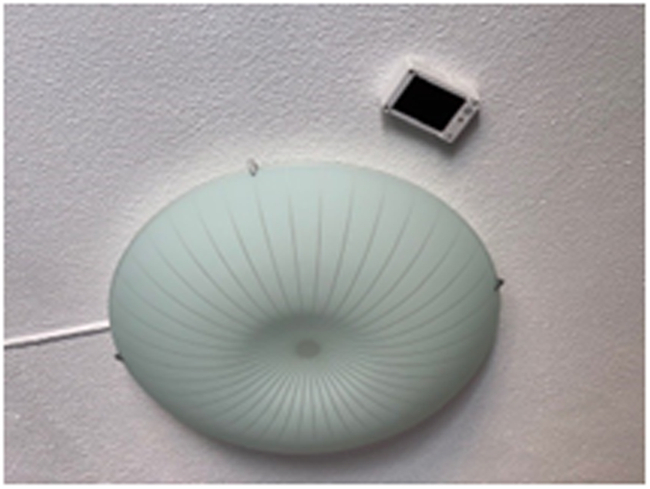
Self-Powered Wireless Sensor.

Self-Powered Wireless Sensors (please see Ruano *et al*.^75^) are used for measuring climate room data, such as air temperature and relative humidity, status (open/close) of doors and windows, walls temperature, light and room movement (Fig. 8). They are Ultra-Low-Power devices and communicate via ISM radio band working on 2.4 GHz or 868 MHz frequencies.

**Fig. 9:**
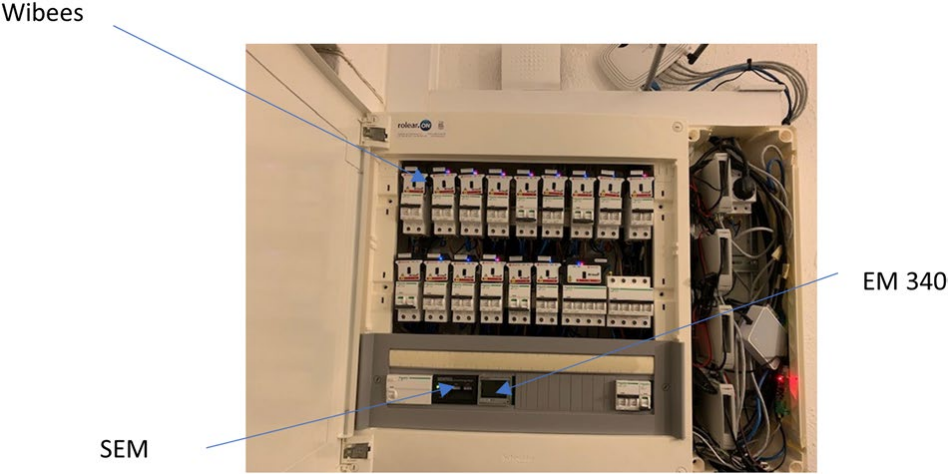
Schneider panel.

Data transmission from/to the measurement devices is available through Gateways and a Technical Network. A technical IP-cabled and a wireless network have been created using a network router, separating the home network from the technical network.

Finally, an IOT platform was created to interactuate with the data acquisition system. For more information on the acquisition system and the IOT platform, please see Ruano *et al*.^76^.

In the three additional houses, only electric consumption is measured. For this reason, in TH2, a Carlo Gavazzi EM340 meter was installed. In MH1 and MH2, Carlo Gavazzi EM112 (one-phase) meters were installed, providing a subset of variables acquired by the EM340.

## Data Records

The data records are available in Zenodo^66^. The datasets are divided in months, starting in January 2020, and ending in February 2023, spanning therefore more than three years. They are Matlab data files, with the format ‘v7’, which can be loaded using the usual ‘*load’* Matlab command. Notice that the use of this format enables the data to be read directly by other languages, such as python, using the function *loadmat* in *scipy.io*.

The sensing devices are categorized in eight categories, and within each category, there might be different appliances.

The variables measured by the Wibeees are shown in Table 2.

**Table 1:** Categories and Devices classifications.

Categories	Device numbers	Comments
1 - Wibees	1–19	See Tables 2, 3
2 – EM340	20,31	See Table 10
3 - Inverter	21	See Tables 4–8
4 – Smart Plugs	22–24, 34	See Table 11
5 – Weather Station	25	See Table 12
6 - SPWS	26–29	See Tables 13–16
7 – Air Conditioner	30	See Table 17
8 – EM 112	32-33	See Table 9

**Table 2:** Variables measured by Wibeees.

Variable	Variable Name	Units
Time basis	dtvec	datetime
Number of Samples	ndtvec	
Voltage	Vvec	Volts
Current	Ivec	Ampere
Frequency	Fvec	Hertz
Active Power	APvec	kW
Reactive Power	RPvec	kVAR
Apparent Power	ApPvec	kVA
Power Factor	PFvec	
Active Energy	AEvec	kWh
Inductive Reactive Energy	IREvec	kVARh
Capacitive Reactive Energy	CREvec	kVAh

All variables are matrices (except *ndtvec*, which is a vector).

There are sixteen monophasic WBs and 1 triphasic. The monophasic WBs range from one to fifteen, and nineteen. The triphasic one ranges from sixteen to eighteen, corresponding to each one of three phases. The most important electric appliances in TH1 are shown in Table 3.

**Table 3:** Wibees and major appliances (please see Fig. 1).

Wibeee	Appliances
1	Alarm
2	Swimming-pool Pump and lights, Garden Appliances
3	Illumination 1^st^ floor
4	Illumination and Plugs Ground Floor (Hall, Garage, Bedroom, Bathroom)
5	Air conditioner Office 1^st^ floor
6	Garage and outside gates
7	Air conditioners B12 and B13
8	Pantry plugs (Thermo-Accumulator, Washing and Drying machines)
9	Kitchen plugs (Dish Washing Machine, Microwave, etc)
10	Air conditioner A14
11	Kitchen plugs (2 fridges, Coffee Machine, etc
12	Living Room plugs (TV, Sound System, etc)
13	Plugs 1^st^ Floor (Computing Equipment, towel heater, ceramic heater)
14	Air conditioner Living Room
15	Illumination Ground Floor (Living room, Dining Room, Kitchen)
16	Burner Stove 1
17	Burner Stove 2
18	Oven
19	Weather Station, Data Acquisition System

The data acquisition of the wibeees is asynchronous. This means that there is a time basis for each device. The different time basis are stored in the matrix *dtvec*. The number of samples for each device is stored in the vector *ndt*. Therefore, if you want to plot the evolution of the phase factor of, let us say, wibeee 6, you should use the Matlab command:

- *plot(dtvec(1:ndtvec(6),6),PFvec(1:ndtvec(6),6)*

There are several variables associated with the inverter/battery. These variables are sampled at a 1 minute rate. They are detailed in Tables 4–8.

**Table 4:** Variables related with the inverter/battery in TH1.

Variable	Variable Name	Units
Time Basis	dtINVvec	datetime
Number of Samples	ndtINVvec	
Active Power (L1)	INVPMAP_L1vec	kW
Active Power (L2)	INVPMAP_L2vec	kW
Active Power (L3)	INVPMAP_L3vec	kW
Total Active Power	INVPMAP_sysvec	kW
Reactive Power (L1)	INVPMRP_L1vec	kVAR
Reactive Power (L2)	INVPMRP_L2vec	kVAR
Reactive Power (L3)	INVPMRP_L3vec	kVAR
Total Reactive Power	INVPMRP_sysvec	kVAR
Apparent Power (L1)	INVPMApP_L1vec	kVA
Apparent Power (L2)	INVPMApP_L2vec	kVA
Apparent Power (L3)	INVPMApP_L3vec	kVA
Total Apparent Power	INVPMApP_sysvec	kVA
Current (L1)	INVPMI_L1vec	Ampere
Current (L2)	INVPMI_L2vec	Ampere
Current (L3)	INVPMI_L3vec	Ampere
Voltage (L1)	INVPMV_L1vec	Volt
Voltage (L2)	INVPMV_L2vec	Volt
Voltage (L3)	INVPMV_L3vec	Volt

All variables are vectors (except *ndtINVvec*, which is a scalar). This table represents grid connection values.

**Table 5:** Variables related with the inverter/battery in TH1.

Variable	Variable Name	Units
DC Power (string 1)	INVDCP_L1vec	kW
DC Power (string 2)	INVDCP_L2vec	kW
DC Power (string 3)	INVDCP_L3vec	kW
DC Power (All)	INVDCP_sysvec	kW
DC Current (string 1)	INVDCI_L1vec	Ampere
DC Current (string 2)	INVDCI_L2vec	Ampere
DC Current (string 3)	INVDCI_L3vec	Ampere
DC Voltage (string 1)	INVDCV_L1vec	Volt
DC Voltage (string 2)	INVDCV_L2vec	Volt
DC Voltage (string 3)	INVDCV_L3vec	Volt

This table represents DC values.

**Table 6:** Variables related with the inverter/battery in TH1.

Variable	Variable Name	Units
State of Charge	INVBASCvec	%
Charge Current	INVBCCvec	Ampere
Discharge Current	INVBDCvec	Ampere
Charge (−)/Discharge(+) Power	INVBCDPvec	kW
Gross Capacity	INVBGCvec	kW
Number of cycles	INVBNCvec	
State of Charge	INVBSCvec	%
Temperature	INVBTvec	°C
Voltage	INVBVvec	Volt

This table represents Battery values.

**Table 7:** Variables related with the inverter/battery in TH1.

Variable	Variable Name	Units
Total DC power (sum of all PV inputs)	INVTotDCpowervec	W
Total AC Charge (AC-side to battery)	INVTotACchargevec	Wh
Total AC discharge energy (battery to grid)	INVTotACdischargevec	Wh
Total DC energy from PV1	INVTotDCPV1vec	Wh
Total DC energy from PV2	INVTotDCPV2vec	Wh
Total DC energy from PV3	INVTotDCPV3vec	Wh
Total DC PV energy (sum of all PV inputs)	INVTotDCPVvec	Wh
Total energy (AC-side to grid)	INVTotACenergyvec	Wh
Total DC charge energy (DC-side to battery)	INVTotDCchargevec	Wh
Total DC discharge energy (DC-side from battery)	INVTotDCdischargevec	Wh

This table presents total values.

**Table 8:** Variables related with the inverter/battery in TH1.

Variable	Variable Name	Units
Active_Power (L1)	INVAP_L1vec	kW
Active_Power (L2)	INVAP_L2vec	kW
Active_Power (L3)	INVAP_L3vec	kW
Total Active_Power	INVAP_sysvec	kW
Current (L1)	INVI_L1vec	Ampére
Current (L2)	INVI_L2vec	Ampére
Current (L3)	INVI_L3vec	Ampére
Voltage (L1)	INVV_L1vec	Volt
Voltage (L2)	INVV_L2vec	Volt
Voltage (L3)	INVV_L3vec	Volt
Total Apparent Power	INVApP_sysvec	kVA
Total Reactive Power	INVApP_sysvec	kVAR
Total Home Consumption Rate	INVCRvec	kW
Power Consumption (from Battery)	INVPCBvec	kW
Power Consumption (from Grid)	INVPCGvec	kW
Power Consumption (from PV)	INVPCPVvec	kW
Energy Consumption (from Battery)	INVECBvec	kWh
Energy Consumption (from Grid)	INVECGvec	kWh
Energy Consumption (from PV)	INVECPVvec	kWh
Total Energy Consumption	INVECPVvec	kWh
Invertor Generation Energy	INVGEvec	kWh
Invertor Generation Power	INVGPvec	kW
Inverter State	INVISvec	
Manager State	INVMSvec	
Power Factor	INVPFvec	%
Power Limit	INVPFvec	
Work Time	INVWTvec	
Daily Yeld	INVYDvec	
Monthly Yeld	INVYMvec	
Yearly Yeld	INVYYvec	
Total Yeld	INVYTvec	

This table shows home and other variables.

There are several variables associated with the EM112 and EM340 meters. These variables are sampled at a one second rate. They are detailed in Table 9 for the monophasic meters, and in Table 10, for the triphasic ones. The variables might be vectors (if only one house is measured in the corresponding period) or matrices (if there are measurements available for the two houses).

**Table 9:** Variables related with home consumption, from EM112 meters.

Variable	Variable Name	Units
Time Basis	dtEM1vec	datetime
Number of Samples	ndtEM1vec	
Voltage	EMVvec	Volt
Current	EMIvec	Ampere
Active Power	EMAPvec	kW
Reactive Power	EMRPvec	kVAR
Apparent Power	EMApPvec	kVA
Power Factor	EMPFvec	%
Frequency	EMF1Pvec	Hertz
Total Active Energy	EMAEP1Pvec	kWh
Partial Active Energy	EMRE1Pvec	kWh
Total Reactive Energy	EMRE1Pvec	kVARh
Partial Reactive Energy	EMREP1Pvec	kVARh
Power Demand	EMDP1Pvec	kW
Peak Power Demand	EMDPP1Pvec	kW

**Table 10:** Variables related with home consumption, from EM340 meters.

Variable	Variable Name	Units
Time Basis	dtEMvec	datetime
Number of Samples	ndtEMvec	
Voltage (L1-L2)	EMVL1_L2vec	Volt
Voltage (L1-N)	EMVL1_Nvec	Volt
Voltage (L3-L1)	EMVL3_L1vec	Volt
Voltage (L3-N)	EMVL3_Nvec	Volt
Voltage (L2-L3)	EMVL2_L3vec	Volt
Voltage (L3-N)	EMVL2_Nvec	Volt
Voltage (L-L)	EMVL_L_sysvec	Volt
Voltage (L-N)	EMVL_N_sysvec	Volt
Current (L1)	EMI_L1vec	Ampere
Current (L2)	EMI_L2vec	Ampere
Current (L3)	EMI_L3vec	Ampere
Active Power (L1)	EMAP_L1vec	kW
Active Power (L2)	EMAP_L2vec	kW
Active Power (L3)	EMAP_L3vec	kW
Total Active Power	EMAP_sysvec	kW
Reactive Power (L1)	EMRP_L1vec	kVAR
Reactive Power (L2)	EMRP_L2vec	kVAR
Reactive Power (L3)	EMRP_L3vec	kVAR
Total Reactive Power	EMRP_sysvec	kVAR
Apparent Power (L1)	EMApP_L1vec	kVA
Apparent Power (L2)	EMApP_L2vec	kVA
Apparent Power (L3)	EMApP_L3vec	kVA
Total Apparent Power	EMApP_sysvec	kVA
Power Factor (L1)	EMPF_L1vec	%
Power Factor (L2)	EMPF_L2vec	%
Power Factor (L3)	EMPF_L1vec	%
Total Power Factor	EMPF_sysvec	%
Frequency	EMFvec	Hertz
Active Energy (L1)	EMAE_L1vec	kWh
Active Energy (L2)	EMAE_L2vec	kWh
Active Energy (L3)	EMAE_L3vec	kWh
Total Active Energy	EMAETvec	kWh
Partial Active Energy	EMAEPvec	kWh
Total Reactive Energy	EMRETPvec	kVARh
Partial Reactive Energy	EMARPvec	kVARh
Power Demand	EMDPvec	kW
Peak Power Demand	EMDPPvec	kW

The maximum number of Smart Plugs existent in TH1 was 4. Data was sampled at one second. The measured variables are represented in Table 11.

**Table 11:** Variables measured by Smart Plugs.

Variable	Variable Name	Units
Time Basis	dtSPvec	datetime
Number of Samples	ndtSPvec	
Voltage	VSPvec	Volts
Current	ISPvec	Ampere
Active Power	APSPvec	kW
Active Energy	AESPvec	kWh
Signal Power	SPRssivec	dbs
On/off	SPOnvec	0/1

All variables are matrices (except *ndtSPvec*, which is a vector).

The Intelligent Weather Station measures data minute by minute. The variables are shown in Table 12.

**Table 12:** Variables measured by the IWS.

Variable	Variable Name	Units
Time Basis	dtWSvec	datetime
Air Temperature	WS_ATvec	°C
Relative Humidity	WS_RHvec	%
Solar Radiation	WS_RADvec	W/m2

All variables are vectors.

The Self-Powered Wireless Sensors measure variables in 4 compartments of TH1: in the first floor, the Hall, Bedrooms 1_2 and 1_4, and in the ground floor, the Lounge (please see Fig. 1). Data is sampled at 1 minute intervals. The measured data is shown in Tables 13–16.

**Table 13:** Variables measured at Hall in the first floor (appliance 26).

Variable	Variable Name	Units
Time Basis	dtH1vec	datetime
Air Temperature	H1_ATvec	°C
Relative Humidity	H1_RHvec	%
Light	H1_Lvec	Lumens

All variables are vectors.

**Table 14:** Variables measured at Bedroom 1_2 in the first floor (appliance 27).

Variable	Variable Name	Units
Time Basis	dtB12vec	datetime
Air Temperature	B12_ATvec	°C
Relative Humidity	B12_RHvec	%
Light	B12_Lvec	Lumens

All variables are vectors.

**Table 15:** Variables measured at Bedroom 1_4 in the first floor (appliance 28).

Variable	Variable Name	Units
Time Basis	dtB14vec	datetime
Air Temperature	B14_ATvec	°C
Wall Temperature	B14_WTvec	°C
Movement	B14_M	%

All variables are vectors.

**Table 16:** Variables measured at Lounge in the ground floor (appliance 29).

Variable	Variable Name	Units
Time Basis	dtLvec	datetime
Air Temperature	L_ATvec	°C
Relative Humidity	L_RHvec	%
Movement	L_M	%

All variables are vectors.

Finally, Table 17 illustrates the variables measured by the Air Conditioner at bedroom 1_4. Data is measured at one minute intervals.

**Table 17:** Variables measured by the AC in bedroom B_14, first floor.

Variable	Variable Name	Units
Time Basis	dtACvec	datetime
Indoor Temperature	AC_ITvec	°C
Reference Temperature	AC_RTvec	°C
Outdoor Temperature	AC_OTvec	°C
Power State (On/Off)	AC_PSvec	0/1
Swing Mode (On/off)	AC_SMvec	0/1
Eco Mode (On/off)	AC_EMvec	0/1
Turbo Mode (On/off)	AC_TMvec	0/1
Operational Mode	AC_SMvec	
Fan Speed	AC_FSvec	%

All variables are vectors.

## Technical Validation

Until now, we have mentioned variables named as ‘***vec’. They are a raw version of the variables ***, with possibly interpolated data (please see below). The time basis for each one of the 34 devices is, as already specified, different from each other, and expressed in each dt***vec variables.

As for processing a single time basis is needed, all variables have been down-sampled to a 5 minutes sample time, where the values for each sample are the mean values of the corresponding variable, during the corresponding five minutes interval. Energy variables have been down-sampled to a one hour interval.

Consider, as an example, the month of August 2020. There, *PFvec* (Phase Factor of the 19 wibeees) has a size of 2,675,237*19, while the averaged version, *PFveccon*, has a size of 8,928*19. The common power time basis is available in the date variable *dtveccon*, and for energy values the common time basis is in *dteneveccon*.

**Fig. 10:**
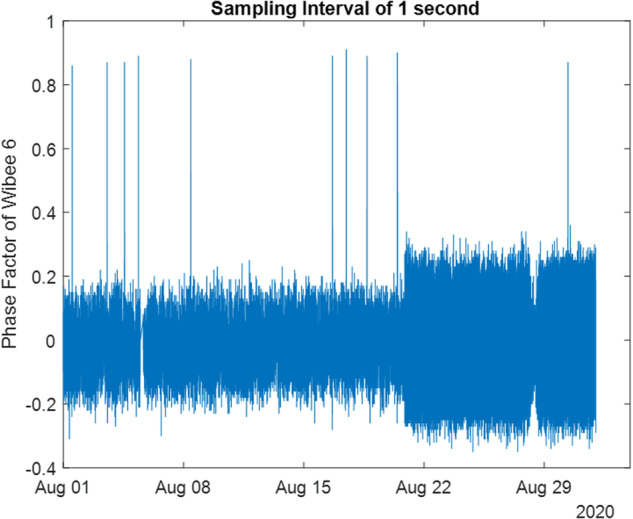
Phase Factor of Wibeee 6, with its own time basis (one second).

**Fig. 11:**
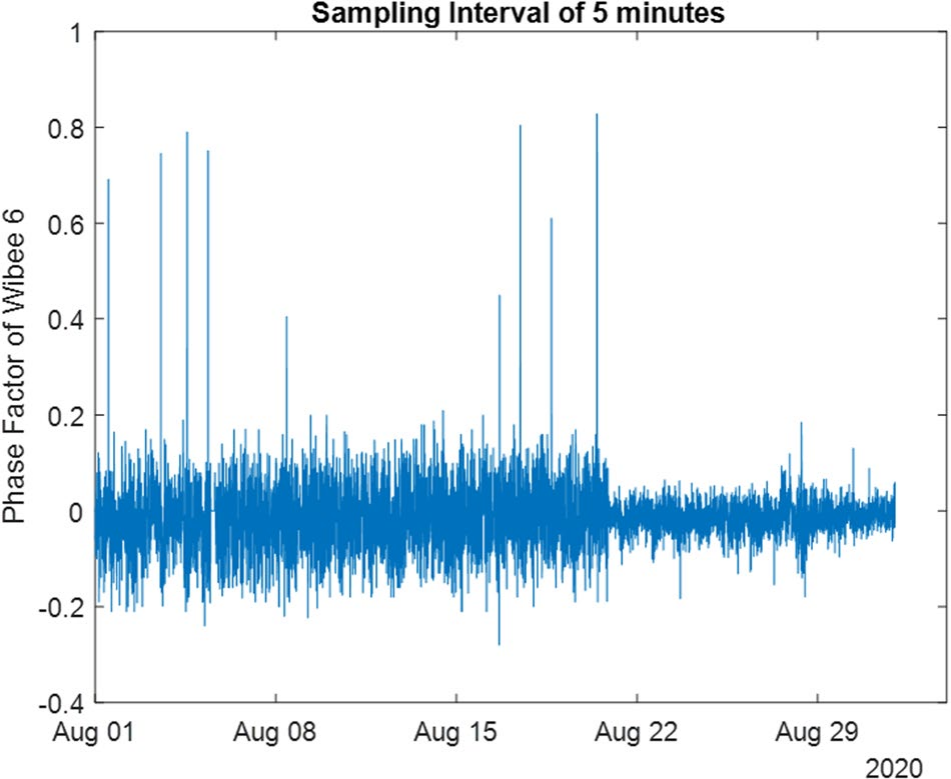
Phase Factor of Wibeee 6, with the common time basis (five minutes).

This way, if you want to plot the evolution of the phase factor of, let us say, wibeee 6, you should use (please see Fig. 10):

*plot(dtvec(1:ndtvec(6),6),PFvec(1:ndtvec(6),6))*

while if you are happy with only the averaged values (please see Fig. 11), you would use:

*plot(dtveccon,PFveccon(:,6))*.

With real-time measured data, there is always the possibility of having missing or invalid data. All measured data is pre-processed, to check for possible gaps. If the number of consecutive missing values is less than seven, the values are interpolated with a moving median scheme; if not they are left as 0 and the period with no data is marked.

Data are also validated. At present only the ranges of temperature, humidity and solar radiation are verified. Valid ranges are:

- Smart Plugs: Current [0 inf]
- WS: AT [−10 50]; RH [0 120]; RAD [0 1500]
- SPWS Hall: AT [−10 50]; RH [0 120]
- SPWS Bed 1_2: AT [−10 50]; RH [0 120]
- SPWS Bed 1_4: AT [−10 50]; RH [0 120]; M [0 100]
- SPWS L: AT [−10 50]; RH [0 120]; M [0 100]
- AC: AC_RT [−10 50]; AC_IT [−10 50]; AC_OT [−10 50]

The information about interpolated data, gaps and faults can be found at the data file with the extension *_stat.mat*. This information can be seen in the following matrices (notice that the categories and device numbers in Table 1 are used here):

- *STEM*, *ENDEM* – matrices with the number of rows equal to the number of appliances, recording the start and the end of periods without data
  For instance, for the same August 2020 month, appliance 29 (the SPWS for the lounge) does not have data between 01-Aug-2021 20:52:54 and 01-Aug-2020 23:42:35, among other gaps.
- *STON*, *ENDON* - start and end samples of the periods with data
  For the same appliance, the first period when there are valid data is between 01-Aug-2020 00:00:36 and 01-Aug-2020 20:52:54
- *nEM*/*nON* - number of periods without data/with data
  For the same appliance, there are 71/72 periods without data/with data
- *inicio/fim -* beginning/end of the data acquisition for each appliance
- *ttotal -* total number of seconds of the specified period of analysis
  Each gap can be inspected with:
- *gaps -* array of records with all the gaps. Their structure is:
  - devices (category of the appliance)
  - num (appliance number)
  - k – sample index for the start of the gap
  - tbeg/tend - time of the start/end of the gap
- *tgap -* total duration (in secs) of the gaps for each appliance
  For appliance 29 and for the same period, for 77 hours, 57 minutes and 50 sec there were no acquired data. This device and device # 27 (SPWS B_12) have a significant percentage of missing data. This does not happen with the other variables (for August 2020 the mean of missing data for the other variables is 16,288 sec and the median 15,411 sec (that is around 0.6% of the total data).
  Faults can be inspected with:
- *tfault* - *information* about the total duration of the faults: array with 7 records for each device group. Each record has the following fields:
  - *num:* number of variables checked for the category
  - *dev:* array of records with the number of appliances in the group which are checked for validity: each record has:
    - *nvars* (number of variables checked)
    - *var* (variable names)
    - *t* (total faulty time for the specified variable).
- *faulttot* – array with records for each fault. It has the following fields:
  - *devices* – category of the appliance
  - *num* – appliance number
  - *var* – variable inspected
  - *kbeg*/*kend* – sample numbers where the fault started/ended
  - *tbeg*/*tend* - time the fault started/ended

For instance, in August 2020 eight faults were recorded. The first was verified for appliance 28, belonging to category 6 (the SPWS for Bedroom 1_4). The fault was verified for the temperature, started in sample 632 and ended in sample 633, or from 03-Aug-2020 13:49:11 to 03-Aug-2020 13:50:18.

- *nsamplesint*/*nsamples* - number of interpolated samples/total number of samples per appliance

For instance, for wibeee 2, the total number of samples was 2,643,997. Among them 161 were interpolated (less than 0.01%).

As explained before, Wibees needed to be calibrated, before being useful. This was done, for each Wibee, using an external instrument measuring electric power, and confronting this value with the value available through the acquisition system. This gave initial factor values, which were subsequently fine-tuned by a phase-by-phase optimization procedure, making use of the Carlo Gavazzi measured data. These multiplying factors, which are used by the Matlab file *extract_quadro_10.m*, are available in the Matlab data file *Factor.mat*. (please see below). It should be noted that this optimization procedure was executed in a monthly basis, to verify if further calibrations were needed. The factor values remained, however, constant throughout the project. .

Apart from small communication problems, there were no anomalies found for the Carlo Gavazzi meters, as well as the for KI and KEM meters. As mentioned before, they were solved by interpolation, if possible, or identified by the detection of gaps.

## Data Availability

All code for the generation of the dataset was written in Matlab R2022 and can be found at https://github.com/aebruano/HEMStoEC. Daily information is received by the data acquisition system in a zipped file, which should be placed in the same directory (denoted as root directory) of the function files. A sample can be found in 2023_06_11_00_00_00.zip. The README and the VARS files provide information about the format of the files enclosed in the zip file. Matlab data is extracted from the unzipped file using the Matlab function extract_quadro_10.m. The command *extract_quadro_10(‘2023_06_11_00_00_00’*) creates a Matlab data file *2023_06_11_00_00_00.mat* inside the *2023_06_11_00_00_0*0 directory. Gaps are identified and data is interpolated using the function Validate_Quadro_4.m. A data file *2023_06_11_00_00_00_cor.mat* is created, again inside the *2023_06_11_00_00_0*0 directory, upon the command *Validate_Quadro_4(‘2023_06_11_00_00_00’,‘2023_06_11_23_23_59’)*.Data with a common time basis is achieved using the Matlab function convert_quadro_10_cor.m. Using the command convert_quadro_10_cor(‘2023_06_11_00_00_00’,‘2023_06_11_23_23_59’,”, minutes(15),hours(1)), the data file *2023_06_11_00_00_00 to 2023_06_11_23_23_59 excl pst 15* *min est 1 hr_cor.mat* is created, this time in the root directory. A matlab file, *Factor.mat*, needs to be placed in the root directory.
